# Bottleneck analysis at district level to illustrate gaps within the district health system in Uganda

**DOI:** 10.1080/16549716.2017.1327256

**Published:** 2017-06-05

**Authors:** Dorcus Kiwanuka Henriksson, Mio Fredriksson, Peter Waiswa, Katarina Selling, Stefan Swartling Peterson

**Affiliations:** ^a^ Institution of International Maternal and Child Health, Department of Women’s and Children’s Health, Uppsala University, Uppsala, Sweden; ^b^ Department of Public Health Sciences, Karolinska Institutet, Stockholm, Sweden; ^c^ Department of Public Health and Caring Sciences, Uppsala University, Uppsala, Sweden; ^d^ Makerere University College of Health Sciences, School of Public Health, Kampala, Uganda

**Keywords:** Tanahashi model, bottleneck analysis, district health systems, maternal and newborn care, supply-side determinants, demand-side determinants

## Abstract

**Background:** Poor quality of care and access to effective and affordable interventions have been attributed to constraints and bottlenecks within and outside the health system. However, there is limited understanding of health system barriers to utilization and delivery of appropriate, high-impact, and cost-effective interventions at the point of service delivery in districts and sub-districts in low-income countries. In this study we illustrate the use of the bottleneck analysis approach, which could be used to identify bottlenecks in service delivery within the district health system.

**Methods:** A modified Tanahashi model with six determinants for effective coverage was used to determine bottlenecks in service provision for maternal and newborn care. The following interventions provided during antenatal care were used as tracer interventions: use of iron and folic acid, intermittent presumptive treatment for malaria, HIV counseling and testing, and syphilis testing. Data from cross-sectional household and health facility surveys in Mayuge and Namayingo districts in Uganda were used in this study.

**Results:** Effective coverage and human resource gaps were identified as the biggest bottlenecks in both districts, with coverage ranging from 0% to 66% for effective coverage and from 46% to 58% for availability of health facility staff. Our findings revealed a similar pattern in bottlenecks in both districts for particular interventions although the districts are functionally independent.

**Conclusion:** The modified Tanahashi model is an analysis tool that can be used to identify bottlenecks to effective coverage within the district health system, for instance, the effective coverage for maternal and newborn care interventions. However, the analysis is highly dependent on the availability of data to populate all six determinants and could benefit from further validation analysis for the causes of bottlenecks identified.

## Background

Poor access, utilization, and quality of care [1,2] account for about two-thirds of maternal and child deaths globally – deaths that are preventable through effective and affordable interventions [3–7]. Due to constraints and bottlenecks both within and outside the health system [8], effective interventions often do not reach the people who need them the most. However, there is limited understanding of health system barriers to delivery and utilization of these affordable and effective interventions in districts and sub-districts in low-income countries [9], where service delivery takes place [8]. Most studies focus on the global and national levels [10,11] where studies have identified barriers within the health system using clinical [12] and patient pathway frameworks [13].

Tanahashi’s concept of health services coverage and evaluation [14] is one of the models that can be used to identify gaps in service delivery. The gap, in this case, refers to the proportion of the target population that does not receive effective coverage [15]. First described in 1978 [14], it displays bottlenecks in the health system with a focus on quality and effectiveness of interventions. The model emphasizes the importance of effective coverage, which is defined as coverage of sufficient quality to reach a defined health impact [14,16,17] and not merely geographic access [18].The model incorporates coverage according to five measures, each reflecting a stage in provision of services, that can be used to assess the potential of a health system to provide effective coverage [15].

In 2002, UNICEF (the United Nations Children’s Fund), the World Health Organization, and the World Bank made some modifications to the Tanahashi model primarily for its use at the national level in low-income countries (LIC) [19] in the Marginal Budgeting for Bottlenecks (MBB) tool. Furthermore, the modified Tanahashi model was used in Papua New Guinea, Nepal, India, Philippines, and Indonesia as part of the Investments Case Framework that supports planning and budgeting [20–22]. It was also used in a field trial in Bangladesh to establish policy opportunities for evidence-based planning for immunization and its limitations [23]. However, although bottleneck analysis is widely used in LIC at national level, there is limited evidence on its use and utility at district level. Therefore, this study aims at illustrating how a modified Tanahashi model can be used to identify bottlenecks in service delivery at the district level. Data for selected maternal and newborn interventions in two rural districts in Uganda were used.

## Methods

### Modified Tanahashi model

**Figure 1.**
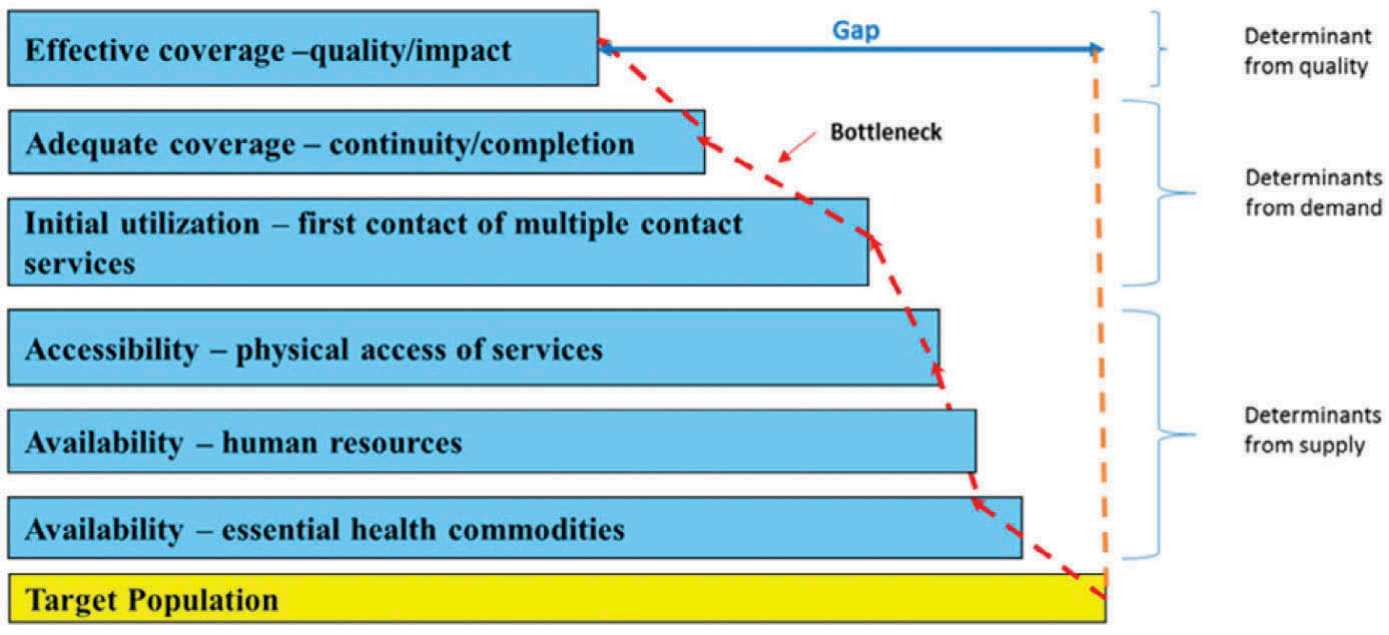
Modified Tanahashi model applied to analyze bottlenecks.

The Tanahashi model used in this study as described earlier was modified for its use in the MBB tool, which was developed to enable LIC at the national level to plan for marginal allocations to health services, cost and budget for these allocations, and access their potential effect on health coverage [19]. One modification to the Tanahashi model still focuses on determinates of effective coverage which is defined as coverage of sufficient quality to reach a defined health impact [14,16,17] and not merely geographic access [18]. Each determinant is analogous to a Tanahashi stage leading towards effective coverage. Another modification was divided, however, the determinant ‘availability’ into the availability of human resources and availability of commodities. This was thought to reflect the types of data that are available and still allow for a stepwise approach to identification of bottlenecks to achieving effective coverage [15]. The modified Tanahashi model therefore has six determinants for effective coverage. The first three determinants – accessibility, availability of human resources, and availability of essential health commodities – are supply-side determinants of the health system, while initial utilization, continuous utilization, and effective coverage focus on the demand side, as illustrated in Figure 1. Supply-side determinants are defined as those factors that influence the health care production function. Demand-side determinants are those that operate at the community, household, and individual levels and are influenced by demand [24]. The six coverage determinants are explained in Table 1. Similar to the original model, the six determinants reflect six distinct aspects of service provision that can be used for a stepwise assessment. Examining the largest differences between each determinant indicates the larger losses of health system effectiveness, thus pointing to those areas of service provision that need to be prioritized. This loss of effectiveness is referred to as a ‘bottleneck’ within the health system [15].

**Table 1.** Definition of coverage determinants used in the Tanahashi model.

Coverage determinants	Definition
Availability of essential health commodities	Refers to availability of health system inputs, for example, medicines and related commodities for maternal and newborn care.
Availability of human resources	Represents availability of staff at health facilities that provide maternal and newborn care services.
Accessibility	Physical accessibility of service delivery points.
Initial utilization	Refers to first contact or use of health services or interventions, for example, first antenatal visit.
Continuous coverage	Refers to the extent to which the full course of contact with the health system required to be effective was achieved, for example, the proportion of women receiving four antenatal contacts.
Effective coverage	Represents the quality of the intervention which is defined as the minimum inputs and processes sufficient to achieve defined health effects.

The three supply-side determinants do not have the same denominator and the presence or absence of one of them does not necessarily affect the presence or absence of the other supply-side determinants, although they tend to be positively correlated, while the demand-side determinants assume a linear relationship.

### Selected interventions

We selected a set of interventions to function as proxies for analyzing health system bottlenecks. After reviewing approximately 190 maternal and child health interventions, Kerber et al. [25] proposed three main channels for their delivery: community-based preventive and health promotion services, outreach services, and clinical and curative services. Due to the specific nature of the delivery channel, it tends to experience similar bottlenecks across interventions. Therefore ‘tracer’ interventions can be selected to serve as proxies to determine bottlenecks for interventions delivered in a similar way. For example, indicators that assess access, availability, initial and continuous utilization, and effective coverage for treatment of pneumonia can be used as proxies to determine bottlenecks for other curative and clinical services, for example, treatment of diarrhea and malaria [26].

The following criteria were used to select interventions: (1) their relevance and appropriateness to maternal and newborn care in Uganda; (2) being internationally recommended for maternal and newborn care; (3) having evidence of measurable impact on outcomes; and (4) availability of data for all six coverage determinants. For this study we chose: (1) use of iron and folic acid supplements to prevent anemia during pregnancy; (2) intermittent presumptive treatment for malaria; (3) HIV counseling and testing, and (4) syphilis testing during antenatal care (ANC) [27–30].

Interventions provided during ANC visits were specifically selected because data were available on these interventions and because good-quality ANC has been documented to improve health outcomes for maternal and newborn care in LIC [31,32]. In Uganda, ANC is provided through clinical services in public health facilities at no cost to the pregnant woman.

### Study design, setting, data collection, and participants

This was a descriptive cross-sectional study using household and facility census surveys. The study was conducted in the two rural districts of Mayuge and Namayingo in the Eastern region of Uganda. The district population is approximately 474,000 and 216,000, respectively [33]. In these districts maternal and newborn care is predominantly provided by public health facilities at different levels. In Uganda, health services are provided through health facilities (including Health Centres [HC] IIs, IIIs, and IVs and hospitals) and Village Health Teams (comprised of community volunteers) [34,35]. Although a lot of progress has been made, maternal and newborn deaths are still unacceptably high in this area with the estimated maternal mortality ratio at 438 per 100,000 live births and an estimated neonatal mortality rate of 23 per 1000 live births [36]. It is, therefore, essential to identify bottlenecks to effective service delivery for maternal and newborn care in order to improve the quality of care in those districts.

Data for this study were collected through household surveys and repeat health facility censuses in Mayuge and Namayingo districts from November 2011–April 2014 [37,38]. This was during the Expanded Quality Management Using Information Power project (EQUIP), which focused on improving the quality of care for maternal and newborn care. Sample size for the project was calculated to estimate coverage of key maternal and newborn interventions with 80% power at the district level [38]. The household surveys used continuous cluster sampling of 10 household clusters using the probability of selection being proportional to the population size. Each cluster had 30 randomly selected households [38]. For the purposes of this study, data from survey interviews with 6513 women who were pregnant 12 months prior to data collection were included (see Table 2).

**Table 2.** Data sources used for the study.

		Public health facilities			
District	Number of women interviewed	HC II	HC III	HC IV	Total
Mayuge	3372	24	4	2	30
Namayingo	3141	16	3	1	20
Total	6513	40	7	3	50

The health facility census was repeated every four months in all government-owned health facilities in the districts. Data from 50 facilities (30 in Mayuge and 20 in Namayingo) were used in this study. These included 20 HC IIs, 7 HC IIIs, and 3 HC IVs (see Table 2). Facility readiness was assessed by interviewing health facility managers and by use of a checklist to determine the routine care and services provided.

### Data analysis and measurements

Data were analyzed using STATA 13 and construction of the bottleneck analysis graphs was done in Excel 2010. Coverage for each determinant was calculated as a proportion of the target population/supply for which a particular determinant was met. See Table 3 for details on the analysis of the coverage levels for each of the determinants and assumptions made.

**Table 3.** Coverage measures used for each of the modified Tanahashi model determinants and assumptions made.

	Interventions			
Determinants	Use of iron and folic acid to prevent anemia during pregnancy	Intermittent presumptive treatment for malaria	HIV counseling and testing	Syphilis test
Accessibility	Average straight line distance from the woman’s household to the nearest public health facility.^2^	Average straight line distance from the woman’s household to the nearest public health facility.^2^	Average straight line distance from the woman’s household to the nearest public health facility.^2^	Average straight line distance from the woman’s household to the nearest public health facility.^2^
*Assumption*	*The facility with the shortest straight line distance from the woman’s household was the ‘nearest’ to her.*			
	*The women sought care from the ‘nearest’ health facility.*			
Availability of human resources	Average proportion of staff employed at the facility on the day of the survey as compared to the Uganda national staffing guidelines.^1^	Average proportion of staff employed at the facility on the day of the survey as compared to the Uganda national staffing guidelines.^1^	Average proportion of staff employed at the facility on the day of the survey as compared to the Uganda national staffing guidelines.^1^	Average proportion of staff employed at the facility on the day of the survey as compared to the Uganda national staffing guidelines.^1^
*Assumption*	*All the staff employed at the facility can provide maternal and newborn care.*			
Availability of essential health commodities	Proportion of times both ferrous sulphate and folic acid or combined ferrous/folate were in stock at the health facility on the day of the facility census.	Proportion of times Sulphadoxine Pyrimethamine was in stock at the health facility on the day of the facility census.	Proportion of health facilities that provide HIV diagnostic services that had HIV test kits in stock on the day of the survey.	Proportion of health facilities that provide syphilis diagnostic services that had syphilis rapid test kits in stock on the day of the survey.
*Assumption*	*That facilities report availability of drugs and diagnostics correctly.*			
Initial utilization	Proportion who took tablets or syrup for 30 or less days.	Proportion of women who attended ANC clinic during the pregnancy.	Proportion of women who got information related to HIV/AIDS and on being tested for the HIV virus.	Proportion of women who attended ANC clinic during the pregnancy.
*Assumption*	*That the women were able to accurately recall how many days they took the medicine and would tell the truth about it.*	*That all the women who received Sulphadoxine Pyrimethamine actually used it as intermittent presumptive treatment of malaria.*	*That the women were able to accurately recall if they were given information related to HIV/AIDS and on being tested for the HIV virus.*	*That the women were able to accurately recall if they attended ANC during that pregnancy.*
		*That the women were able to accurately recall how many doses they were given.*		
Continuous utilization	Proportion who took tablets or syrup for 31–89 days.	Proportion of women who received one dose of malaria prevention medicine.	Proportion of women who gave blood for any test during pregnancy.	Proportion of women who gave blood for testing during the pregnancy.
*Assumption*	*That the women were able to accurately recall how many days they took the medicine and would tell the truth about it.*	*That all the women who received Sulphadoxine Pyrimethamine actually used it as intermittent presumptive*	*That the women were able to accurately recall if they gave blood for any test during that pregnancy.*	*That the women would accurately recall if they gave blood for any test during that pregnancy.*
		*treatment of malaria*. *That the women were able to accurately recall how many doses they were given.*		
Effective coverage	Proportion who took tablets or syrup for 90 days.	Proportion of women who received at least two doses of malaria prevention medicine.	Proportion of women who gave blood for testing during the pregnancy, who received HIV test results.	Proportion of women who gave blood for testing during the pregnancy, who received syphilis test results.
*Assumption*	*That the women were able to accurately recall how many days they took the medicine and would tell the truth about it.*	*That all the women who received Sulphadoxine Pyrimethamine actually used it as intermittent presumptive treatment of malaria*. *That the women were able to accurately recall how many doses they were given.*	*That the women were able to accurately recall if they received HIV test results.*	*That the women would accurately recall if they received syphilis test results.*

## Results

**Figure 2.**
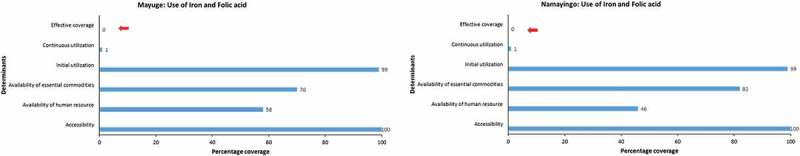
Use of iron and folic acid during pregnancy.

**Figure 3.**
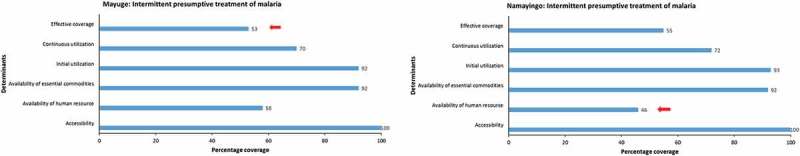
Intermittent presumptive treatment of malaria.

**Figure 4.**
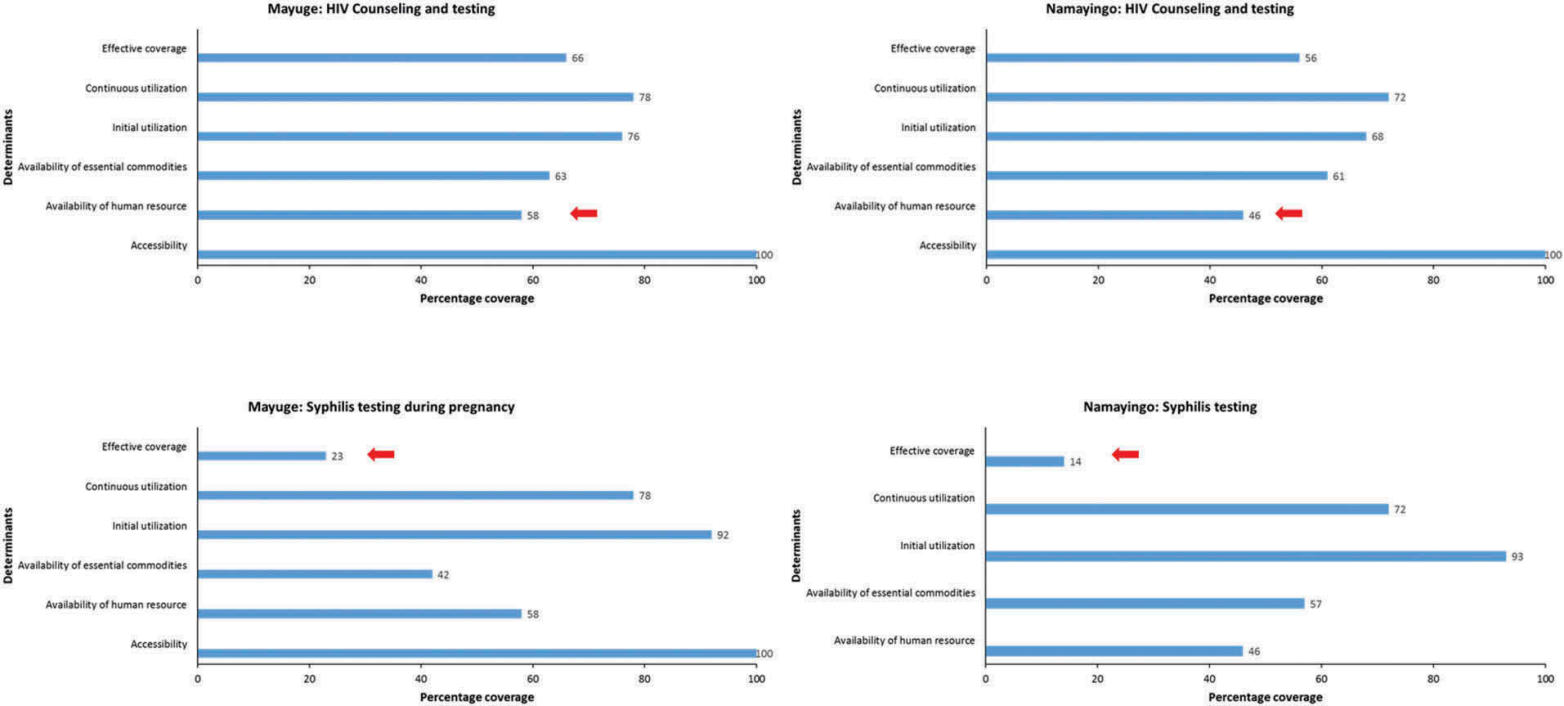
HIV counseling and testing and syphilis testing during pregnancy.

Data analysis included household surveys with 6513 women and census surveys in 50 facilities (see Table 2). Levels of coverage for each determinant and the bottlenecks to service delivery of the selected interventions were identified as illustrated by the bar graphs (see Figures 2–4). Details on each determinant and assumptions made are presented (see Table 3). Ninety-two percent of women in Mayuge and 93% in Namayingo district attended ANC at least once. In contrast, only 40% and 38% attended ANC four or more times in Mayuge and Namayingo districts, respectively. This was slightly lower than the national average of 48% [36].

### Accessibility

Geographical access to a health facility in both districts was 100% as all the interviewed women lived at most 5 km from a health facility, which is the nationally recommended distance [35]. The mean distance to a health facility was on average 2.2 km – 80% of which were HC IIs in both districts. HC IIs are the first level of the formal public health sector and offer only outpatient care [34].

### Availability of human resources

The human resource coverage in both districts was below the national minimum level of at least 69% of posts filled [35], at 58% and 46% in Mayuge and Namayingo districts, respectively. This was the biggest bottleneck for HIV counseling and testing services in both districts and for intermittent presumptive treatment for malaria in Namayingo district (see Figures 3 and 4).

### The use of iron and folic acid supplements during pregnancy

Of the women who did visit a health facility and received iron and folic acid, almost all reported taking it for at least 30 days (initial utilization), whereas only 1% reported taking it for 31–89 days (continuous utilization). None reported taking it for the nationally recommended 90 days (effective coverage) (see Figure 2). Thus the biggest bottleneck was one on the demand side as the pregnant women didn’t take the iron and folic acid for the recommended 90 days [39].

### Intermittent presumptive treatment of malaria for pregnant women (ITPp)

Although Sulphadoxine-Pyrimethamine, the drug used for intermittent presumptive treatment of malaria, was available at the facilities about 92% of the time, and about 92% of women reported having attended ANC at least once (initial utilization), only about 70% of the women reported having received one dose (continuous utilization) and 53% and 55% reported having received at least two doses (effective coverage) in Mayuge and Namayingo districts, respectively (see Figure 3). These results show that even when the drug was available at the health facility, it was not always offered to the pregnant women during ANC visits. This implies that in this case, the bottleneck was the practice of the service providers.

### HIV counseling and testing during pregnancy

One of the bottlenecks in both districts was the availability of commodities. Of all the facilities that routinely provided HIV testing services only about 62% had test kits available on the day of the survey (see Figure 4). Another bottleneck was found in the practice of the service providers as women reported having given blood for HIV testing (continuous utilization) without having been counseled (initial utilization) about HIV/AIDS.

### Syphilis testing during pregnancy

The majority of women who gave blood for testing reported not having received any test results for syphilis. Only 23% in Mayuge district and 14% in Namayingo district reported having received test results (effective coverage). This, coupled with the lack of syphilis rapid test kits, accounted for the greatest bottlenecks to providing the syphilis test during pregnancy (see Figure 4).

## Discussion

Applying the modified Tanahashi model at district level in Uganda, we identified bottlenecks across four tracer interventions for maternal and newborn care. Effective coverage and human resource gaps were the biggest bottlenecks in the two selected districts. In the discussion, we address, firstly, the most important empirical findings related to the tracer interventions, and secondly, more general observations from using the modified Tanahashi model at the district level in a LIC.

Out of the eight largest potential bottlenecks (four from each district), five were related to the last step, ‘effective coverage’, which is similar to findings from studies in e.g. Tanzania documenting very low effective coverage for maternal and newborn care [40,41]. For instance, effective coverage was the main bottleneck in relation to the use of iron and folic acid and syphilis testing during pregnancy, implying low adherence to taking the pills and syphilis testing protocols, respectively. Similar low adherence to iron and folic acid supplements during pregnancy has previously been documented in e.g. Indonesia [42] and Senegal [43,44], and as this is a demand-side determinant, it calls for increased awareness among pregnant women as well as the community at large in order to support long-term use to improve women’s iron levels during pregnancy. It is also important to increase awareness about the importance of attending ANC at least four times during the pregnancy, as is recommended by the national guidelines [39].

However, bottlenecks were also found within the supply side: e.g. inadequate supplies of commodities and drugs such as iron and folic acid [45], syphilis test kits, and HIV test kits, despite some of the drugs being cheap and the significant attention that has been given to HIV-related care in recent years in Uganda [45–48]. These findings are similar to findings in Tanzania [49], where restricted availability of medicines and medical supplies led to poor-quality obstetric care. Here solutions lie on the ‘supply side’, requiring further investigation in the local context as to whether the issue is at facility level (e.g. not ordering), district level (e.g. not distributing), or national level (e.g. procurement and distribution).

Human resource shortage was a major finding, the share of staff posts filled being 58% and 46% in Mayuge and Namayingo districts, respectively. This was a bottleneck in particular for HIV counseling and testing, and for intermittent presumptive treatment of malaria in Namayingo district. Generally, our coverage results for human resources speak to shortages of staff at health facilities which will not only affect service delivery for maternal and newborn care, but will have implications for the clinical and curative services within the health system as well [50,51], since ANC is provided through the same channel in Uganda [39]. However, the practice of the service providers affected the quality of services. As shown in our results for the cases of intermittent presumptive treatment of malaria, HIV counseling and testing, and syphilis testing, even when all commodities are in place health workers may fail to apply them appropriately. Similar results related to the practice of service providers have been documented in Tanzania [40,52]. Therefore, the human resource gaps in terms of absolute numbers and the practice of service providers should both be addressed to enable better functioning of the health systems.

At the district level, we show that bottleneck analysis using the modified Tanahashi model combining household data and health facility data can be done. Dividing the determinant ‘availability’ into ‘availability of human resources’ and availability of essential commodities’ provides an opportunity to focus on these areas that are essential to strengthening health systems especially in Low and Middle-Income Countries (LMIC). The graphs also indicate the bottlenecks on both the demand and supply sides of the health system, as well as the magnitude of the gap that should be filled to achieve effective coverage for a particular intervention. These graphs provide an opportunity for the district managers to critically question the causes of the identified bottlenecks which may have otherwise been missed. Furthermore, by choosing appropriate tracer interventions – used as proxies for the functioning of the health system – bottlenecks relevant to the district health system as a whole can be identified [15,19,53].

In our study, the four selected tracer interventions – and the identified bottlenecks of effective coverage and human resources – suggest that the local health system has problems in delivering quality care even when it comes to relatively cheap drugs and tests with proven and large-scale effects. Furthermore, it indicates that the health of women and children during pregnancy can be improved by a combination of strategies. These could involve educating women and the community at large about the beneficial effects of ANC interventions as well as educating and increasing the number of staff. The modified Tanahashi model for bottleneck analysis can in this way be used to measure the quality of care and develop district-specific solutions that combine supply- and demand-side factors. However, bottleneck analysis should not be considered an end in itself but rather a step to facilitate the prioritization of interventions in the planning process, as these can be affected by many other factors such as governance and leadership, financial resources, decision space, and the role of other stakeholders [54–56]. The so-called ‘root cause analysis’, which engages local managers to study their own data and identify their own constraints and solutions, should follow the bottleneck analysis process. This is being tested in a separate study [57].

We found a similar bottleneck pattern in both districts, although actual coverage levels of the determinants for the tracer interventions varied between the districts. These similarities might be related to the geographic proximity of the districts [58] – both are located in the Eastern Region of Uganda, but are functionally independent – or due to similar health system shortages throughout the country. Another study has, however, shown different bottleneck patterns at the district level for similar interventions, in Tanzania [40]. Thus, we argue that even if overall patterns are similar, there is value in carrying out the bottleneck analysis in each district as there may be important coverage level differences as well as contextual differences that call for different solutions.

There are limitations to the use of bottleneck analysis at district level. Data availability may limit the feasibility if not all six determinants can be populated, requiring special surveys e.g. Lot Quality Assurance Surveys (LQAS) [59]. We recommend efforts to improve routine data collection and validation to enable routine use of bottleneck analysis.

Bottleneck analysis, as we have used it here, depends exclusively on quantitative information. This has limitations. Some indicators may also depend on locally set benchmarks – e.g. living 5 km or less away from a health facility, for geographical accessibility – but nevertheless may not mean functional access in some districts. Furthermore, the analysis would be enriched by employing qualitative methods of data collection with service providers, users of health services, and community members.

Bottleneck analysis as we conducted it in this study considers two health system building blocks: health workforce and access to essential medicines, and uses the building block of service delivery [51] as a way of assessing the function of the whole health system. The approach therefore leaves out the three building blocks of leadership/governance, health information systems, and financing, which are all known to be important for health system strengthening [51,60]. It also does not take into account the broader aspects of health and wellbeing, for example public health, prevention, and the role of other stakeholders that are all important to the health system.

## Methodological considerations

This study was conducted in only two districts which makes the results not easily generalizable, although these districts are very similar to most rural districts in Uganda. Data used to populate the demand-side determinants depended on whether women accurately recalled [61] and reported [62] their encounter with the health system during their last pregnancy. As secondary data were used in this study, data collected could not adequately establish some of the demand-side bottlenecks for IPTp; however, it was able to point to possible bottlenecks within provider practice. The demand-side determinants for HIV counseling and testing and syphilis testing could be attributed to either the service provider practice, as the service provider should request the pregnant woman to give blood for testing and offer her the test results, or the behavior of the woman who needs to consent to giving blood and accept to receive the test results. However, the analysis is able to identify a bottleneck that needs to be addressed.

## Conclusion

The modified Tanahashi model is an analysis tool that can be used to identify bottlenecks to effective coverage within the district health system, for instance, the effective coverage of maternal and newborn care interventions. However, the analysis is highly dependent on the availability of data to populate all six determinants and could benefit from further validation analysis for the causes of bottlenecks identified. Data collection tools and methods at the district level may need to be improved to facilitate bottleneck analysis. The bottleneck analysis tool cannot be used in isolation but is one of the tools that can inform the prioritization of interventions and improve the planning process in LIC like Uganda which could improve quality of care and the district health system.

## Paper context

Poor access, utilization, and quality of care explain almost two-thirds of maternal and child deaths globally, which deaths could be prevented through effective and affordable interventions. However, there is limited understanding of bottlenecks and constraints both within and outside the health system that hinder delivery and utilization of these interventions at the point of service delivery in districts in low-income countries. Bottleneck analysis drawing on the modified Tanahashi model of health services coverage is one way of identifying these bottlenecks.
